# The Editor's Letter-Box

**Published:** 1913-08-09

**Authors:** 


					Th Q ? [Copy.]
? Society for the State Registration of Trained Nurses,
he following resolution was carried nem. con. at the
-^niual Meeting of the Society for the State Registration
^ Trained Nurses, held at the Medical Society's Rooms,
Chandos Street, Cavendish Square, London, W., on
*ric%, July 18, 1913 :
Resolution.
tli Annual Meeting of Members of the Society for
:,.? State Registration of Trained Nurses desires to record
Ys Protest against the practice of the Committee of the
^ ?ndon Hospital in sending out nurses to private cases for
?ain at the end of two years' training, because it is
j Ccjn?mically unsound that they should compete with nurses
, ?\ding certificates of three or more years' consecutive
a in hospital wards, and because, until such time as
Aurses Registration Act is in force, the public cannot
now that the members of the London Hospital private
^rsing staff, for whose services they are charged full fees,
e not required to attain the almost universally accepted
a!1dard of three years' training before certification."

				

